# Network pharmacological analysis, Mendelian randomization analysis and animal experimental reveal the mechanism of Wenxin Granules in inhibiting heart failure after myocardial infarction: apoptosis and inflammation

**DOI:** 10.3389/fcvm.2025.1714639

**Published:** 2026-01-14

**Authors:** Fuyuan Zhang, Baohua Li, Ruikang Liu, Yiying Liu, Xuanchun Huang, Jun Li

**Affiliations:** 1 Cardiovascular Department, Guang'anmen Hospital, China Academy of Chinese Medical Sciences, Beijing, China; 2 Postdoctoral Station of China Academy of Chinese Medical Sciences, Beijing, China; 3 Eye Hospital, China Academy of Chinese Medical Sciences, Beijing, China

**Keywords:** apoptosis, heart failure, inflammation, MAPK3, myocardial infarction, Wenxin Granules

## Abstract

**Background:**

Apoptosis and inflammation are the key pathological mechanisms of heart failure after myocardial infarction (MI-HF). Wenxin Granules (WXG), an effective compound that has been used clinically for more than 20 years, could improve cardiac function, lipids and blood rheology in patients with MI and delay the occurrence of HF. However, the exact mechanism is still unclear.

**Methods:**

MI-HF mice model were established by permanent ligation of the left anterior descending, and the protective effects of WXG on cardiac function and fibrosis were evaluated by Elisa, echocardiography, Masson staining and HE staining. The core components of WXG and its targets and mechanisms of action in MI-HF were clarified by UPLC-MS/MS, network pharmacology and bioinformatics. The association of potential genes with HF was further clarified genetically using Mendelian Randomization Analysis (MR). Molecular docking was utilized to clarify the docking energies between the core components of WXG and the key pathogenic targets of MI-HF. Immunofluorescence, Tunel staining, Elisa, Quantitative real-time polymerase chain reaction (RT-qPCR) and Western blot (WB) were used to evaluate cardiomyocyte apoptosis and inflammatory response, and to validate key proteins on the MAPK pathway and its downstream effect proteins.

**Results:**

The animal experiments showed that WXG significantly improved cardiac function, inhibited myocardial fibrosis, inhibited cardiomyocyte apoptosis and reduced the expression of inflammatory factors in MI-HF mice. Network pharmacology and bioinformatics analyses revealed that WXG may exert its cardioprotective effects through the MAPK signaling pathway. MR further confirmed the high correlation between the apoptotic protein MAPK3 and HF. Molecular docking results showed that Astragaloside IV, Paeoniflorin, Liquiritin, Albiflorin, Ononin, and Pratensein 7-O-beta-D-glucopyranoside, the core components of WXG, were well docked to key pathogenic targets of MI-HF. WB, RT-qPCR and Elisa results showed that pro-apoptotic proteins and pro-inflammatory factors were significantly elevated in the Model group, which was inhibited by WXG.

**Conclusion:**

WXG may reduce inflammatory response and enhance cardioprotection and anti-fibrosis by inhibiting the expression of MAPK signaling pathway and its downstream effect proteins. The cardioprotective effects of WXG may be attributed to its core components, including Astragaloside IV, Paeoniflorin, Liquiritin, Albiflorin, Pratensein 7-O-beta-D-glucopyranoside and Ononin.

## Graphical Abstract

**Figure:**
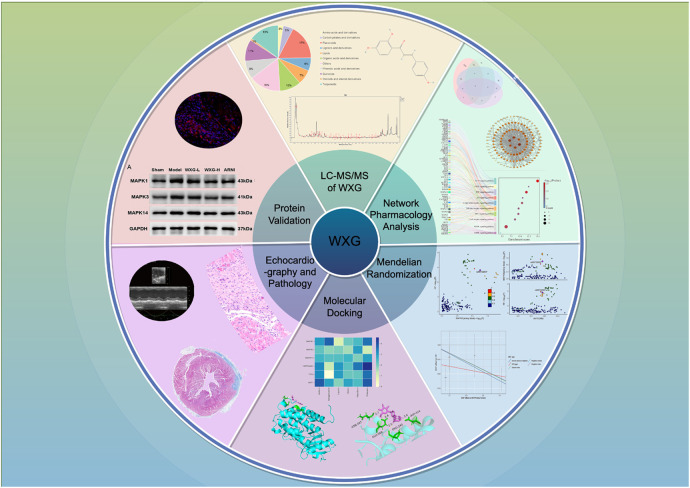
Network pharmacological snalysis, mendelian randomization analysis, and animal experiments reveal the mechanism of Wenxin Granules in inhibiting heart failure following myocardial infarction: apoptosis and inflammation.

## Highlights

1. High expression of MAPK3 may be a key factor in MI-HF.
2. WXG could reduce inflammatory response and inhibit cardiomyocytes apoptosis.
3. WXG could inhibite the MAPK signaling pathway and its downstream proteins.

## 1 Introduction

Cardiovascular diseases are one of the most important health hazards, causing a significant medical and economic burden on society. Ischemic cardiomyopathies, mainly myocardial infarction (MI), account for approximately 51% of cardiovascular disease deaths (1). In recent years, the incidence of MI has been generally increasing and becoming younger (2). Although percutaneous coronary intervention (PCI) has significantly reduced the mortality rate of MI, the incidence of heart failure after MI has increased, which significantly increases the long-term risk of death (3).

Apoptosis and inflammation are crucial factors of MI and heart failure (HF) after MI (MI-HF). After MI, a complex inflammatory response is triggered involving multiple immune cells and inflammatory factors. The balance between pro-inflammatory and anti-inflammatory processes significantly affects cellular function within myocardial tissue. A persistent inflammatory response can lead to ventricular remodeling and complications, and early resolution of inflammation can result in favorable healing outcome (4, 5). Apoptosis is the predominant form of cardiomyocyte death in the early stages of MI and one of the most striking features of cardiac injury in patients with MI is the death of a large number of cardiomyocytes due to the lack of oxygen and energy supply (6). Cardiomyocyte apoptosis occurs in the central and marginal regions of the infarct, and continued apoptosis leads to progressive loss of cardiomyocytes, destruction of intracellular collagen scaffolds, and thinning of the ventricular wall, ultimately leading to ventricular remodeling and the development of heart failure (7). Therefore, it's crucial to develop new effective therapeutic strategies and drugs to improve the inflammatory storm and cardiomyocyte apoptosis to suppress MI-HF.

Chinese Medicine shows unique advantages in the prevention of MI-HF. WXG is an effective formula consisting of 11 herbs condensed from our nearly 30 years of clinical practice and has been granted a Chinese invention patent (No. ZL202210358477.1) (Table 1). Clinical studies have found that WXG is not only effective in improving the symptoms of angina pectoris and ischemic electrocardiogram, but also significantly improves blood rheology and blood lipids (8). However, the mechanism of action of WXG is uncertain. Therefore, this study intends to excavate the potential therapeutic targets of WXG through Liquid chromatography-mass spectrometry (LC-MS/MS), network pharmacology, molecular docking and Mendelian Randomization analysis (MR). Animal experiments were also conducted to verify the effects of anti-inflammatory and anti-apoptotic of WXG, with a view to bringing new treatment for MI-HF (Graphical abstract).

**Table 1:** Specific ingredients of WXG.

Herbal name	Latin scientific name	Plant part	Weight ratio (g)
Dangshen	*Codonopsis pilosula* (Franch.) Nannf*.*	Roots	15
Huangqi	*Astragalus membranaeus* (Fisch.) Bge*.*	Roots	30
Rougui	*Cinnamomum cassia* Presl*.*	Tree bark	3
Chishao	*Paeonia lactiflora* Pall*.*	Roots	12
Chuanxiong	*Ligusticum chuanxiong* Hort*.*	Roots	10
Jianghuang	* Curcuma longa* Linn*.*	Roots	9
Dahuang	* Rheum officinale* Baill.	Roots	6
Ezhu	* Curcuma zedoaria* (Christm.) Rosc*.*	Roots	9
Gualou	* Trichosanthes kirilowii* Maxim*.*	Fruits	12
Xixin	* Asarum sieboldii* Miq*.*	Roots	3
Gancao	* Glycyrrhiza uralensis* Fisch*.*	Roots	10

## 2 Materials and methods

### 2.1 Identification of active ingredients of WXG *in vitro* and *in vivo*

#### 2.1.1 Preparation of WXG

10 g WXG (Sichuan New Green Pharmaceutical Technology Development Co., LTD) was dissolved in 20 mL deionized water and vortexed for 10 min. The supernatant was collected after centrifugation at 12,000 rpm for 10 min. The final WXG extract was filtered through a 0.22 μm microporous membrane filter and used for LC-MS detection.

#### 2.1.2 Collection of WXG components in blood

Twelve male SPF-grade SD rats, weighing 180–220 g, were randomized to treatment or control group, six in each group. The treatment group was gavaged WXG (10.71 g/kg) every 12 h for 3 days. The control group was provided with distilled water following the same administration protocol. The rats were anesthetized at 0.5, 1, 1.5, 2, 3 and 4 h after the last gavage respectively, and blood sample was taken from the abdominal aorta. Blood sample was centrifuged at 3,000 rpm for 15 min at 4 °C and the supernatant was extracted. Then the supernatant was inactivated by heating in a water bath at 56 °C for 30 min, dispensed and frozen at −80 °C for analysis.

#### 2.1.3 LC-MS/MS of WXG

The analytes were detected and analyzed by ultra performance liquid chromatography tandem Fourier transform mass spectrometry (UHPLC-Q Exactive system) from Thermo Fisher Scientific. Chromatographic separation was accomplished using Milford's C18 column. The column size was 100 mm × 2.1 mm with a particle size of 1.7 um. The mobile phase consisted of an aqueous solution with 0.1% formic acid and acetonitrile water as the organic phase. The temperature of the column oven was set at 40 °C, and the injection volume of each sample was precisely 3 uL. The samples were electrospray ionization (ESI), and the mass spectral signals were collected in positive and negative ion scanning modes respectively. The resolution of the mass analyzer (Full MS) was 70,000, and the scanning type was set at 70–10,500 m/z. The Sheath gas flow rate was 50 arb and the Aux gas flow rate was 13 arb. The capillary temperature was set at 320 °C, and the collision energy was set to 20%, 40% and 60%.

Following LC-MS/MS analysis of WXG's chemical constituents, the following data processing steps were performed: Raw mass spectrometry data underwent peak identification, peak alignment, and baseline correction using Progenesis QI software (Waters Corporation), with noise peaks excluded. Major chemical constituents in WXG were identified by comparing retention times with reference standards, precise molecular weights from primary MS, and fragment ion information from secondary MS, ombined with database searches in MassBank, HMDB, and others. Constituents were statistically categorized by chemical class chemical category such as phenolic acids, saponins, flavonoids, and so on. Semi-quantitative analysis employed relative peak area normalization to calculate the relative percentage content of each component. The total peak area of all identified components is set as 100%. The relative content (%) of a single component = (Extracted ion peak area of that component/Total peak area of all identified components)  × 100%. The relative percentage content within the same chemical category is the sum of the relative contents of all components within that category.

### 2.2 Identification of potential targets for the treatment of MI-HF with WXG

The predicted targets and specific components of WXG were obtained through the TCMSP database (https://www.tcmsp-e.com/tcmsp.php) and Herb database (http://herb.ac.cn/). The screening parameters for the drugs were set as OB ≥ 30%, DL ≥ 0.18. In the GeneCards Database (https://www.genecards.org/), “myocardial infarction” and “heart failure” were used as the main search term, and the relevance score was limited to greater than 10 to obtain the disease targets of MI-HF. Further, we searched for disease targets of MI-HF in Therapeutic Target Database (https://db.idrblab.net/ttd/), PharmGKB Database (https://www.pharmgkb.org/), and Online Mendelian Inheritance in Man (https://www.omim.org/). All the disease targets of MI-HF abtained above were removed duplicates and intersected. Morevoer, the potential targets of WXG for the treatment of MI-HF were obtained through the intersection of the drug target and the disease target. And venn figure was drew by R software.

### 2.3 Protein-Protein interaction (PPI) network construction and enrichment analysis

In order to construct PPI Network, we submitted the identified similar genes above to String online database (https://string-db.org/). Cytoscape 3.7.2 software equipped with cytoNCA plugin was used to screen out the core targets of WXG in the treatment of MI-HF. At the same time, the confidence level of each node and the degree of correlation between the nodes are analyzed. Further, Gene Ontology (GO) enrichment and Kyoto Encyclopedia of Genes and Genomes (KEGG) enrichment of cross-targets were conducted by R software to explorate the potential signaling pathway of WXG in the treatment of MI-HF.

### 2.4 Mendelian randomization analysis

To further clarify the correlation between genes and HF from a genetic perspective, we conducted a series of MR. Summary statistics from genome-wide association studies (GWAS) for HF were obtained from the FinnGen consortium (release R12; https://www.finngen.fi/en) (9). Expression quantitative trait locus (eQTL) data were derived from the Genotype-Tissue Expression (GTEx) project (https://www.gtexportal.org) and the eQTLGen consortium (https://eqtlgen.org/), whereas protein quantitative trait locus (pQTL) data were sourced from the Fenland study (10–12).

Table 2 summarizes the GWAS datasets included in the analysis. SNPs strongly associated with eQTL were selected as instrumental variables (IVs). To ensure independence, SNPs in linkage disequilibrium were pruned (*P* > 5 × 10^−8^, *r*^2^ > 0.001, window size = 10,000 kb). Weak IVs (*F* < 10) were excluded, and heterogeneity and horizontal pleiotropy were assessed. To minimize bias from reverse causation, Steiger filtering was applied. Colocalization analysis was performed on two-sample MR results derived from eQTL data, with cis-region defined as ±100 kb around each gene. A posterior probability of shared causality (PPH4 > 0.8) was considered evidence of a common variant influencing both gene expression and HF. Genes passing this threshold were further evaluated using summary-data-based MR (SMR) and the heterogeneity in dependent instruments (HEIDI) test, with SNPs located within ±500 kb of the gene used as IVs. All analyses were conducted in RStudio using the TwoSampleMR and MR-PRESSO packages.

**Table 2:** Data sources in mendelian randomization analyse.

Datasets	Sample	People	Consortium	Download site
The cis-eQTL in blood	31,684	European	eQTLGen	https://eqtlgen.org/
	670	European	GTEx	https://gtexportal.org/home/datasets.
The cis-eQTL in atrial appendage	372	European	GTEx	https://gtexportal.org/home/datasets.
The cis-eQTL in left ventricl	386	European	GTEx	https://gtexportal.org/home/datasets.
The cis-eQTL in artery aorta	387	European	GTEx	https://gtexportal.org/home/datasets.
The cis-eQTL in artery coronary	213	European	GTEx	https://gtexportal.org/home/datasets.
The cis-eQTL in artery tibial	584	European	GTEx	https://gtexportal.org/home/datasets.
Cis-pQTL in blood	4,907	European	Ferkingstad et al. (12)	PMID: 34857953
HF	42,081	European	Henry et al. (9)	PMID: 40038546

### 2.5 Molecular docking and molecular dynamics simulations

Firstly, the crystal structure of the core target was downloaded from the PDB Database (https://www.rcsb.org/), prioritizing high-resolution structures. Subsequently, operations such as removing water molecules, adding hydrogen atoms, and determining atomic rigid structures were performed in the Autodock, with the results saved in pbdqt format. The sdf files of the major components of WXG were obtained from the PubChem database (https://pubchem.ncbi.nlm.nih.gov) and converted to mol2 structure using OpenBabel 2.4.1. Furthermore, the target protein in pdbqt format and the small molecule in mol2 format were simultaneously opened in Autodock. A grid box fully enclosing both molecules was selected, and molecular docking was performed using Autodock Vina with Python scripts. Finally, we visualized the results using PyMOL software. In addition, we calculated Vina scores, with lower Vina values indicating a higher affinity between the receptor and ligand. In the presence of Vina less than or equal to −5, the main ingredients of WXG were considered to bind the target effectively.

### 2.6 Animals

50 male C57 BL/6J mice (8–9 weeks old, 22 ± 24 g) were provided by Beijing Vital River Laboratory Animal Technology Co, Ltd. The experimental mice were adaptive fed for a week in SPF-grade animal laboratory with free intake of food and water. The animal laboratory is kept at a constant temperature of 24 °C with 12 h of alternating light and darkness. This experiment was approved by the Ethics Committee of Guang'anmen Hospital, China Academy of Traditional Chinese Medicine (IACUC-GAMH-2025-038-01). We ensure that the manipulation of animals for feedding, model establishment, pharmacological interventions, and specimen sampling in our experiments is in accordance with the principles of animal kindness.

#### 2.6.1 Preparation of drugs and reagents

WXG were prepared and supplied by Sichuan New Green Pharmaceutical Technology Development Co.,LTD. Sacubitril Valsartan Sodium (ARNI) Tablets were supplied by Novartis Singapore Pharmaceutical Manufacturing Private.Ltd. MAPK1, MAPK3, MAPK14, TP53, HSP90AA1, AKT1, Bcl2, Bax, and Caspase3 antibody were purchased from Affinity Biosciences and the batch number were as follows: BF8004, BF8004, AF3019, DF7238, BF0084, BF8005, AF6139, AF0120 and AF6311. The enhanced chemiluminescence (batch number: 34096) was purchased from Thermo Fisher Scientific. The Rnase-free water was purchased from Beijing Solarbio Science & Technology Co.,Ltd. The Reverse transcription kit (batch number: AG11728) was purchased from Accurate Biotechnology (Hunan) CO.,LTD. The interleukin (IL)-1β, IL-6, IL-17, tumour necrosis factor a (TNF-a) and IL-10, Fibroblast Growth Factor 2 (FGF2), Transforming Growth Factor-beta 1 (TGF-*β*1) and N-terminal pro-brain natriuretic peptide (NT-proBNP) Elisa kit were purchased from Beijing Zhichao Weiye Biotechnology Co.,LTD and the batch number were as follows: ZC-137505, ZC-137519, ZC-137493, ZC-142680, ZC-138183, ZC-138193, ZC-137558 and ZC-140218.

#### 2.6.2 MI-HF model establishment

The MI-HF mouse model was established by permanent ligation of the left anterior descending (LAD). After one week of adaptive feeding, the mice were anesthetized with 3% sodium pentobarbital at a dose of 80 mg/kg by intraperitoneal injection. The mouse chest and axilla were shaved with a mouse shaver and the surgical area was sterilized with iodine and 75% ethanol. Then the trachea was intubated for mechanical ventilation, and chest rise and fall in line with the ventilator frequency indicated successful intubation. The mice were placed in the right lateral recumbent position, and the chest was opened between the third and fourth ribs to fully expose the heart, which was ligated with a 6.0 suture through the LAD 2 mm below the left auricle. After completion of ligation, the chest was closed with a 6.0 suture, and the tracheal tube was removed after respiration and cardiac rhythm were stabilized. Modeling was considered successful when the ligated area and lower segments turned grayish white and ST-segment elevation was demonstrated on electrocardiographic monitoring.

#### 2.6.3 Grouping and treatment

WXG and Sacubitril Valsartan Sodium Tablets were proportionally dissolved in saline to prepare ARNI solution (5.2 mg/mL), WXG High Dose (WXG-H) solution (1.55 g/mL) and WXG Low Dose (WXG-L) solution (0.77 g/mL), respectively. After the MI-HF mouse model established, 50 mice were randomly divided into the Sham Group, the Model Group, ARNI Group (52 mg/kg), WXG-H Group (15.47 g/kg) and WXG-L Group (7.74 g/kg), with 10 mice in each group (12). The Sham Group and Model Group were given saline 0.2 mL/d by gavage. WXG-H Group and WXG-L Group were given WXG-H solution or WXG-L solution 0.2 mL/d by gavage, respectively. ARNI Group were given ARNI solution 0.2 mL/d by gavage. All mice were treated for 4 weeks.

#### 2.6.4 Cardiac function assessed by echocardiography

We used echocardiography to assess cardiac function and various key parameters of mice. The mice were anesthetized intraperitoneally with 3% sodium pentobarbital and their chests were depilated with depilatory agents. The mice were immobilized in a belly-up position on a operating desktop maintained at 40 °C. Cardiac function was assessed via short-axis M-mode echocardiography with measurements averaged across three consecutive cardiac cycles. M-mode images were utilized to evaluate the left ventricular function through measurements of wall thickness and chamber dimensions at the level of the papillary muscles. Key echocardiographic parameters were quantified including: left ventricle ejection fraction (LVEF), Left ventricle fractional shortening (LVFS), left ventricular anterior wall thickness in diastolic (LVAWd), left ventricular anterior wall thickness in systole (LVAWs), left ventricle posterior wall thickness in diastole (LVPWd), left ventricle posterior wall thickness in systole (LVPWs), diastolic left ventricle internal diameter (LVIDd), and systolic left ventricle internal diameter (LVIDs). Both heart rates and body temperature were continuous monitored throughout the scanning procedure.

#### 2.6.5 Pathological examination and immunofluorescence

The thoracic cavity of mice was opened after anesthetized. The hearts were removed and rinsed in saline and placed in 4% Paraformaldehyde phosphate buffer for 48 h. They were paraffin-embedded and sectioned after complete decalcification. Furthermore, We performed HE staining, Masson staining, Tunel staining, immunofluorescence of MAPK3 and observed under the light microscope and photographed.

#### 2.6.6 Serum Elisa assay

Four weeks after treatment, the mice were anesthetized with 3% pentobarbital sodium to obtain blood sample. Then the blood sample was centrifuged at 3,000 rpm for 15 min, and the supernatant was taken in a clean centrifuge tube. Moreover, we detected the NT-proBNP, TGF-β1, FGF2, IL-1β, IL-6, IL-10, IL-17, and TNF-a levels according to the kit instructions.

#### 2.6.7 Western blot

The myocardial tissue of mice were collected, and RIPA buffer containing protease inhibitors was used to dissociate the ankle homogenates and lyse the cells. The protein concentration was determined, and protein electrophoresis was performed using 10% SDS–PAGE gels. After that, the separated proteins were transferred to PVDF membranes and blocked at room temperature with 5% nonfat milk. Primary antibodies were used to detect the corresponding proteins. After washing 3 times with TBST, the membranes were incubated with secondary antibodies. GAPDH served as an internal reference.

#### 2.6.8 RT-qPCR

Total RNA was extracted from myocardial tissue of mice using TRIzol reagent. RNA was reverse transcribed into c DNA using a reverse transcription kit according to the instructions. RNA was reverse transcribed into cDNA using a reverse transcription kit according to the instructions. The mRNAs of key genes were then detected on a real-time fluorescent quantitative PCR system using SYBR Green PCR premix. The reaction conditions were: 94 °C initial step for 2 min, 94 °C for 10 s, 60 °C for 1 min, and 72 °C for 30 s for 40 cycles. The quantitative results were compared with GAPDH as the reference mRNA using 2-ΔΔCT. The primer sequences of the target genes are shown in Table 3.

**Table 3:** qRCR primer sequence.

Gene	Direction	Base sequence	Primer length/bp
MAPK1	Forward	5′-TTTGCTTTCTCTCCCGCACA-3′	181
	Reverse	5′-GGGCTCATCACTTGGGTCAT-3′	
MAPK3	Forward	5′-AACCCAAACAAGCGCATCAC-3′	152
	Reverse	5′-ATCAACTCCTTCAGCCGCTC-3′	
MAPK14	Forward	5′-TGCCCGAACGATACCAGAAC-3′	150
	Reverse	5′-GGTAGGTCCTTTTGGCGTGA-3′	
TP53	Forward	5′-ATATCAGCCTCGAGCTCCCT-3′	108
	Reverse	5′-GCAACAGATCGTCCATGCAG-3′	
HSP90AA1	Forward	5′-CCTGACGGACCCCAGTAAAC-3′	90
	Reverse	5′-TCCACAATGGTCAGGGTTCG-3′	
AKT1	Forward	5′-ATGAGCGACGTGGCTATTGTGAAG-3′	24
	Reverse	5′-TCAGTCTGGTGCACAACTCCGGAT-3′	
GAPDH	Forward	5′-AGGTCGGTGTGAACGGATTTG-3′	95
	Reverse	5′-GGGGTCGTTGATGGCAACA-3′	

### 2.7 Statistical methods

The data were presented as mean ± SDs and analyzed using GraphPad Prism 10.0. ONE WAY ANOVA was used to identify different groups. *P* < 0.05 was considered to be statistically significant.

## 3 Results

### 3.1 Compositional analysis of WXG *in vitro* and *in vivo*

**Figure 1:**
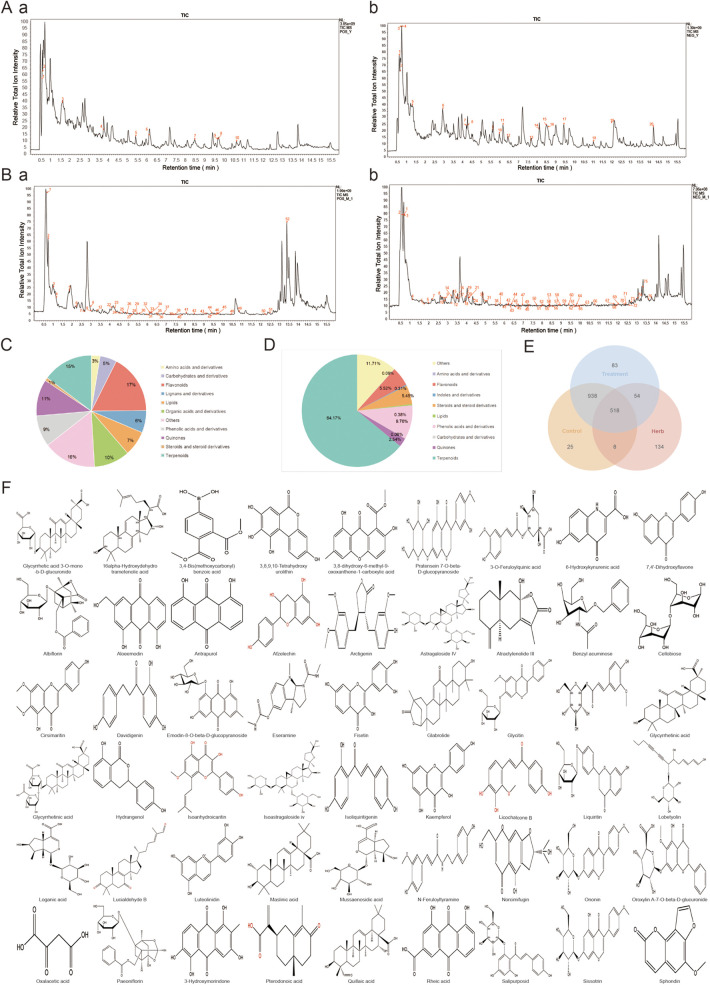
Analysis of the *in vitro* material basis and blood-absorbed components of WXG. **(A)** Total ion chromatogram of WXG in positive ion mode **(a)** and negative ion mode **(b)** of LC-MS/MS. **(B)** Total ion chromatogram of Drug-Containing Serum of WXG in positive ion mode **(a)** and negative ion mode **(b)** of LC-MS/MS. **(C)** Ingredient classification of WXG. **(D)** Ingredient classification of Drug-Containing Serum of WXG. **(E)** Venn intersection analysis between *in-vitro* material composition of WXG, Drug-Containing Serum and blank serum. **(F)** 2D structure of 54 major components.

The positive and negative ion scans of WXG were performed under optimized chromatographic and mass spectrometric conditions (Figure 1A). A total of 714 compounds were eventually identified (Supplementary Table S1), including Amino acids and derivatives 2.6%, Carbohydrates and derivatives 4.91%, Flavonoids 17.33%, Lignans and derivatives 6.44%, Lipids 7.28%, Organic acids and derivatives 10.5%, Phenolic acids and derivatives 9.07%, Quinones 10.59%, Steroids and steroid derivatives 0.97%, Terpenoids 14.82% and Others 15.52% (Figure 1C). Good separation and mass spectral response were obtained for each peak in WXG medicated serum (Figure 1B). A total of 128 compounds were eventually identified (Supplementary Table S2), including Terpenoids 64.14%, Steroids and steroid derivatives 5.45%, Quinones 2.54%, Phenolic acids and derivatives 9.76%, Lipids 0.38%, Indoles and derivatives 0.31%, Flavonoids 5.52%, Carbohydrates and derivatives 0.06%, Amino acids and derivatives 0.09%, Alkaloids and derivatives 0.05% and Others 11.7% (Figure 1D). Further, the comprehensive analysis of original active substances of WXG, WXG medicated serum and control serum were performed. The results showed a total of 54 compounds identified as core components of WXG, which were detected in WXG and WXG medicated serum without in control serum (Figures 1E,F).

### 3.2 Identification of targets and signal pathway of MI-HF for the treatment with WXG

**Figure 2:**
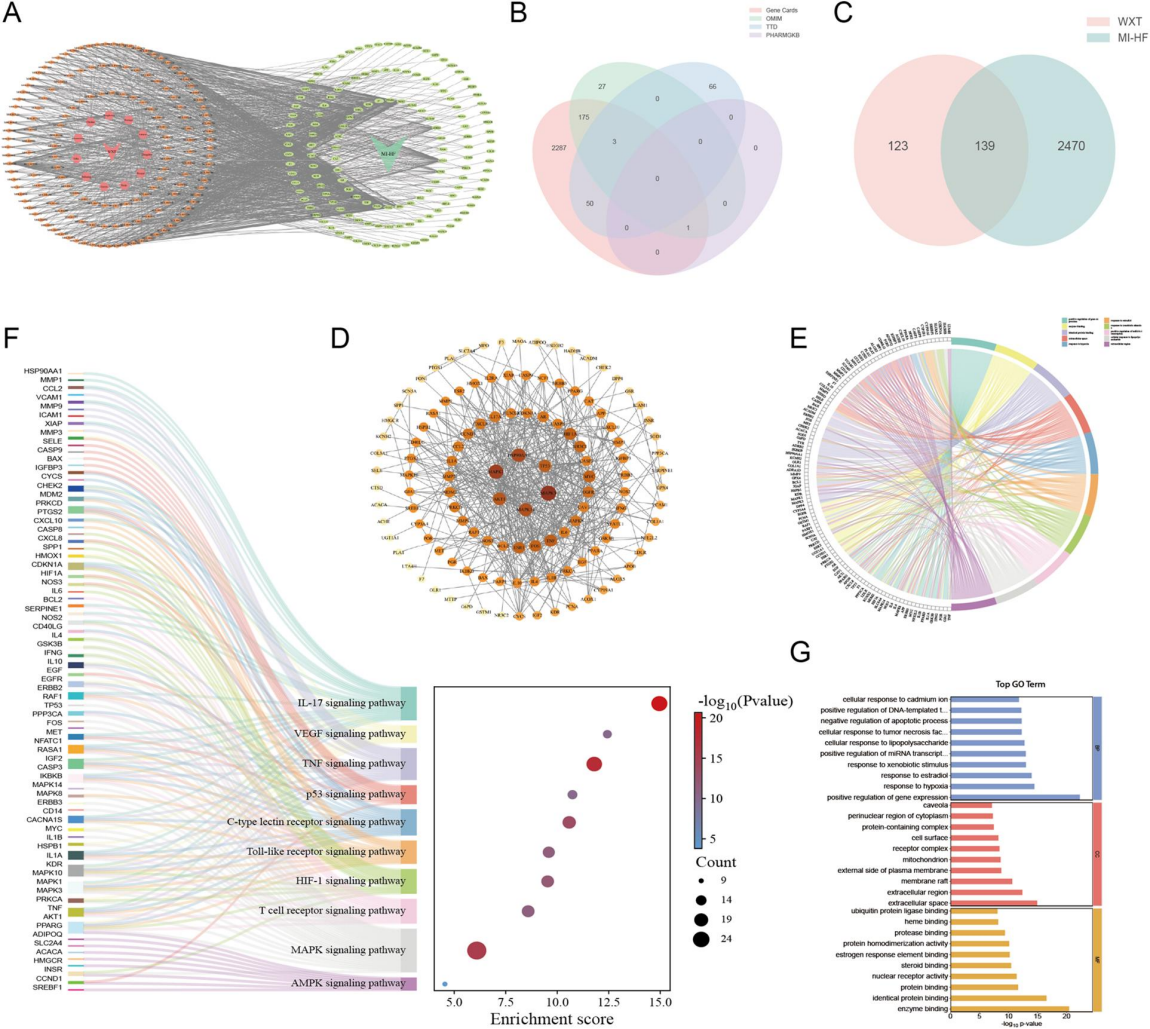
Network pharmacology analysis and enrichment analysis. **(A)** Potential targets for WXG in the treatment of MI-HF. **(B)** Venn intersection analysis of disease targets with MI-HF. **(C)** Venn intersection analysis of common targets of WXG and MI-HF. **(D)** Protein-protein interactions analysis. **(E,F)** Top 10 terms of KEGG analysis. **(G)** GO enrichment analysis (BP, MF, and CC top 10 terms).

Components and therapeutic targets of WXG were obtained from the TCMSP database, which yielded a total of 262 relevant targets after removing duplicates (Figure 2A). Disease targets of MI-HF were retrieved from GeneCards Database, Online Mendelian Inheritance in Man, Therapeutic Target Database and PharmGKB Database. A total of 2,609 targets were obtained after de-duplication and merging (Figure 2B). By combining the related targets of WXG and MI-HF to take the intersection and plotting the VENN figure, 139 duplicate targets were identified as candidate targets. This suggests that WXG may exert a therapeutic effect on MI-HF through these targets (Figure 2C). To investigate the potential interactions of 139 targets, protein-protein interactions (PPI) analysis were performed. A PPI network was generated which consists of 139 nodes and 278 edges by uploading these gene data to the STRING database (https://cn.string-db.org/). The PPI network showing that the top six core targets were MAPK3, MAPK1, HSP90AA1, MAPK14, AKT1 and TP53 by screening the values of Betweenness, Closeness, Degree, Eigenvector and LAC in Cytoscape Software (Figure 2D).

To uncover the function and biological pathways of differentially expressed genes (DEGs), KEGG and GO pathway analyses were conducted. KEGG pathway analysis depicted that pathogenic genes of MI-HF were principally involved in MAPK signaling pathway, IL-17 signaling pathway, VEGF signaling pathway, TNF signaling pathway, p53 signaling pathway, C-type lectin receptor signaling pathway, Toll-like receptor signaling pathway, HIF-1 signaling pathway, T cell receptor signaling pathway and AMPK signaling pathway (Figures 2E,F). GO analysis exhibited that the DEGs were mainly associated with positive regulation of gene expression, enzyme binding, identical protein binding, extracellular space, response to hypoxia, response to estradiol, response to xenobiotic stimulus, positive regulation of miRNA transcription, cellular response to lipopolysaccharide, extracellular region. At the same time, the top 10 terms of Molecular Function, Biological Process and Cellular Components were displayed (Figure 2G).

### 3.3 MR clarifies the correlation between MAPK3 and HF

**Figure 3:**
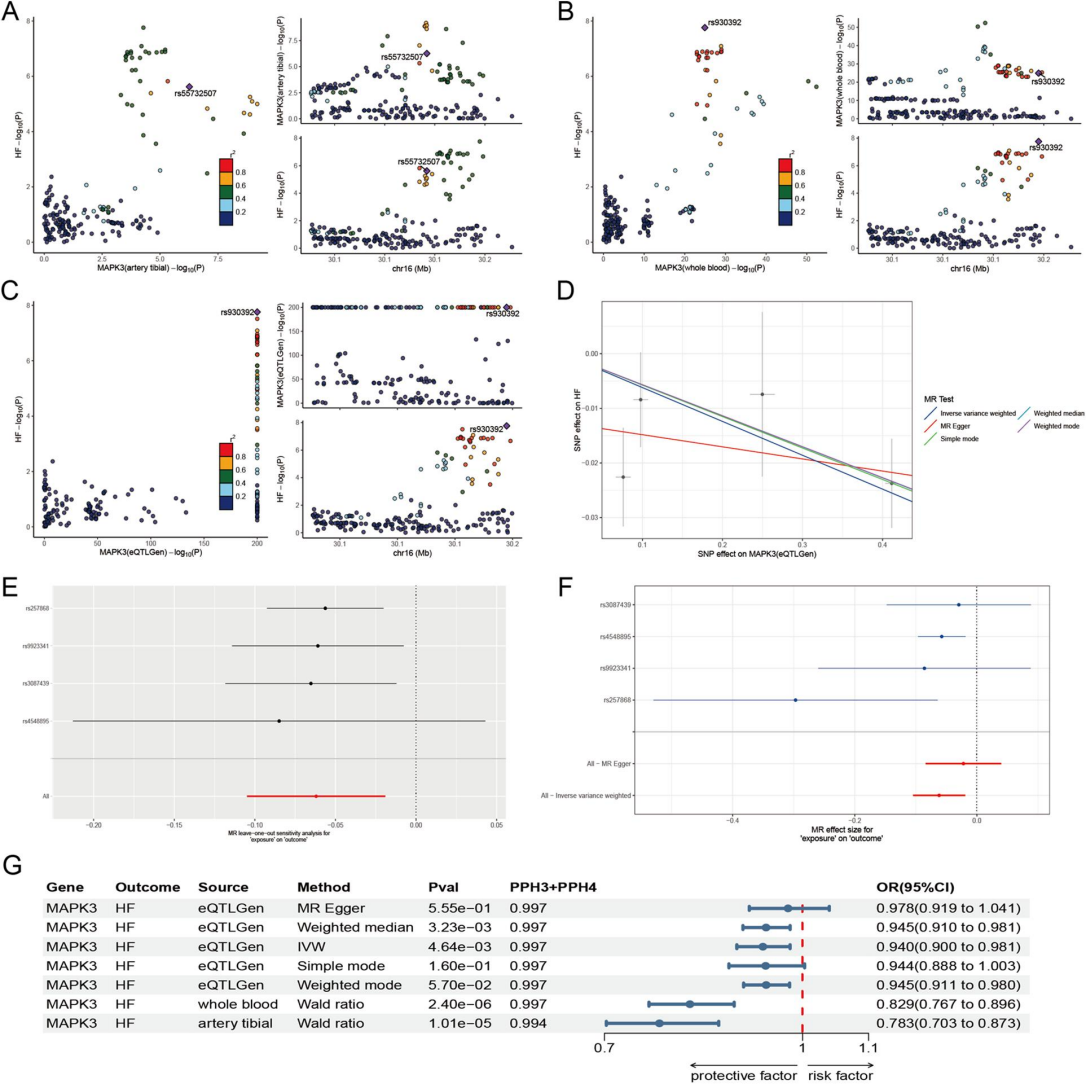
Molecular docking diagram. **(A)** Docking energy heatmap of top 6 components with top 6 targets. **(B–L)** Visualization results of molecular docking between selected components and targets [**(B)** Albiflorin with MAPK3; **(C)** Astragaloside IV with MAPK3; **(D)** Liquiritin with MAPK3; **(E)** Paeoniflorin with MAPK3; **(F)** Pratensein with MAPK3; **(G)** Ononin with MAPK3; **(H)** Liquiritin with MAPK1; **(I)** Paeoniflorin with MAPK14; **(J)** Astragaloside IV with HSP90AA1; **(K)** Astragaloside IV with TP53; **(L)** Liquiritin with AKT1].

To further clarify the correlation between genes and HF from a genetic perspective, we conducted a series of MR. The results demonstrated that the expression of MAPK3 is significantly associated with HF across multiple tissues. In whole blood, the association were observed in both the GTEx dataset (*P* = 2.40 × 10^−6^; OR = 0.829; 95% CI: 0.767–0.896) and the eQTLGen consortium (*P* = 4.64 × 10^−5^; OR = 0.940; 95% CI: 0.900–0.981). A similar association was identified in tibial nerve tissue (*P* = 1.00 × 10^−5^; OR = 0.783; 95% CI: 0.703–0.873) (Figures 3A–G) (Supplementary Tables S3, S4). At the protein level, MAPK3 remained significantly associated with HF in SMR analysis (*P* < 0.05) and showed no evidence of heterogeneity in the HEIDI test (*P* > 0.05), indicating that the observed association is likely driven by a shared causal variant (Supplementary Table S5).

### 3.4 Molecular docking

**Figure 4:**
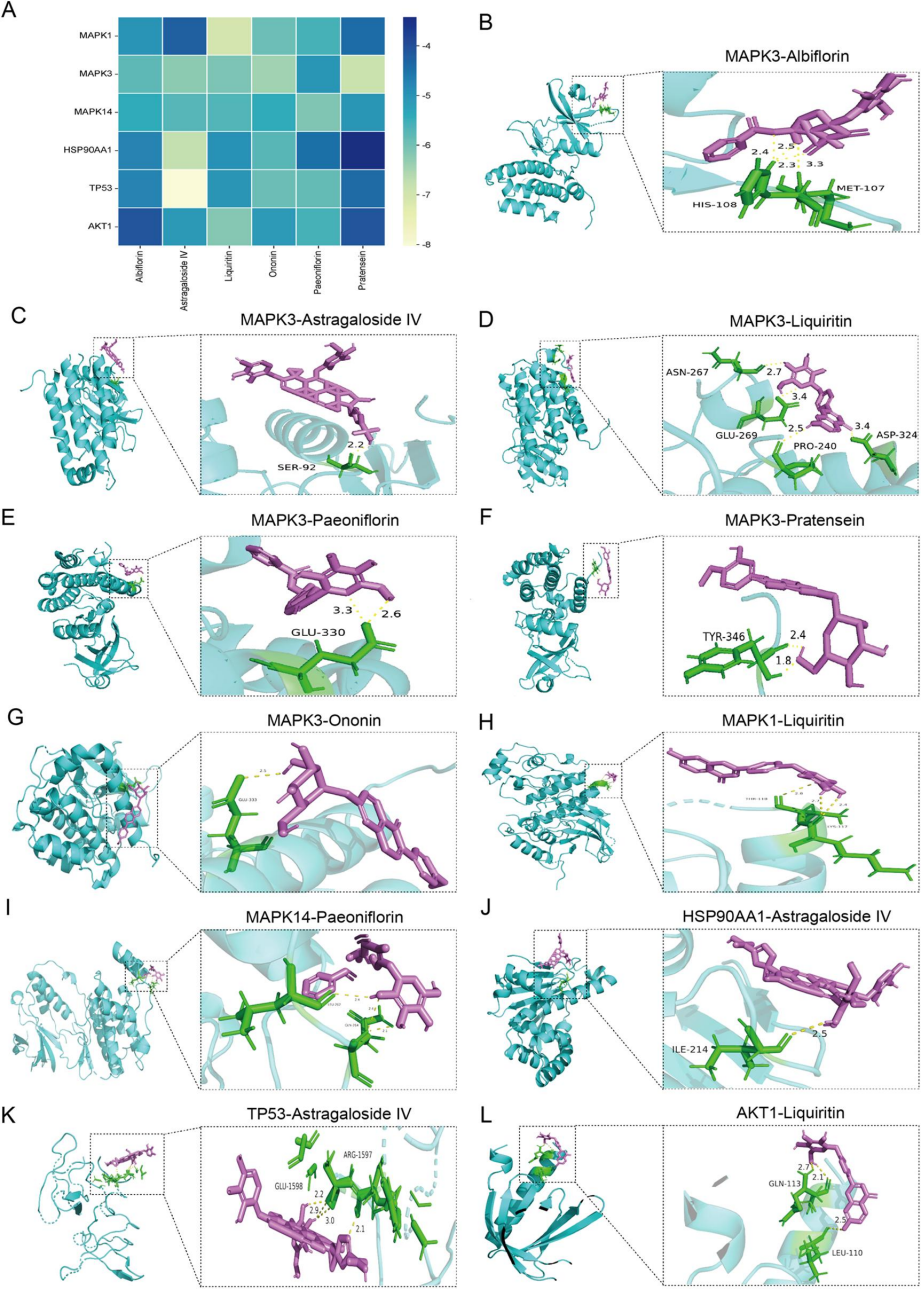
The results of Mendelian randomization analyse. **(A–C)** Co-localization site maps for the expression of MAPK3 in tibial artery **(A)**, whole blood **(B)**, and eQTLGen **(C)**, with HF as the outcome. **(D)** Scatter plot depicting the association between the expression of MAPK3 (eQTLGen) and HF. **(E)** Leave-one-out analysis based on eQTLGen data to evaluate the robustness of the MAPK3-HF association. **(F)** Forest plot of effect estimates for individual genetic instruments linking expression of MAPK3 (eQTLGen) to HF. **(G)** Summary forest plot integrating significant associations from two-sample Mendelian randomization and co-localization analyses across multiple tissues.

Based on the analysis of the *in vitro* ingredient and ingredient of Drug-Containing Serum, Albiflorin, Astragaloside IV, Liquiritin, Paeoniflorin, Pratensein 7-O-beta-D-glucopyranoside, and Ononin are the main active compounds in the treatment of MI-HF with WXG. The interactions of these components with top 6 targets of MI-HF were explored by molecular docking to understand their binding affinities. Lower binding energy indicates stronger binding ability. Docking results showed that all these components bind better to MAPK1, MAPK3, MAPK14, HSP90AA1, TP53 and AKT1 (Figure 4A). The PDB-ID of these 6 target proteins were 4QTA, 7NRB, 2FST, 3O0I, 2G3R, 1UNQ. We present the results of the better binding (Figures 4B–L).

### 3.5 WXG significantly improves cardiac dysfunction of MI-HF mice

**Figure 5:**
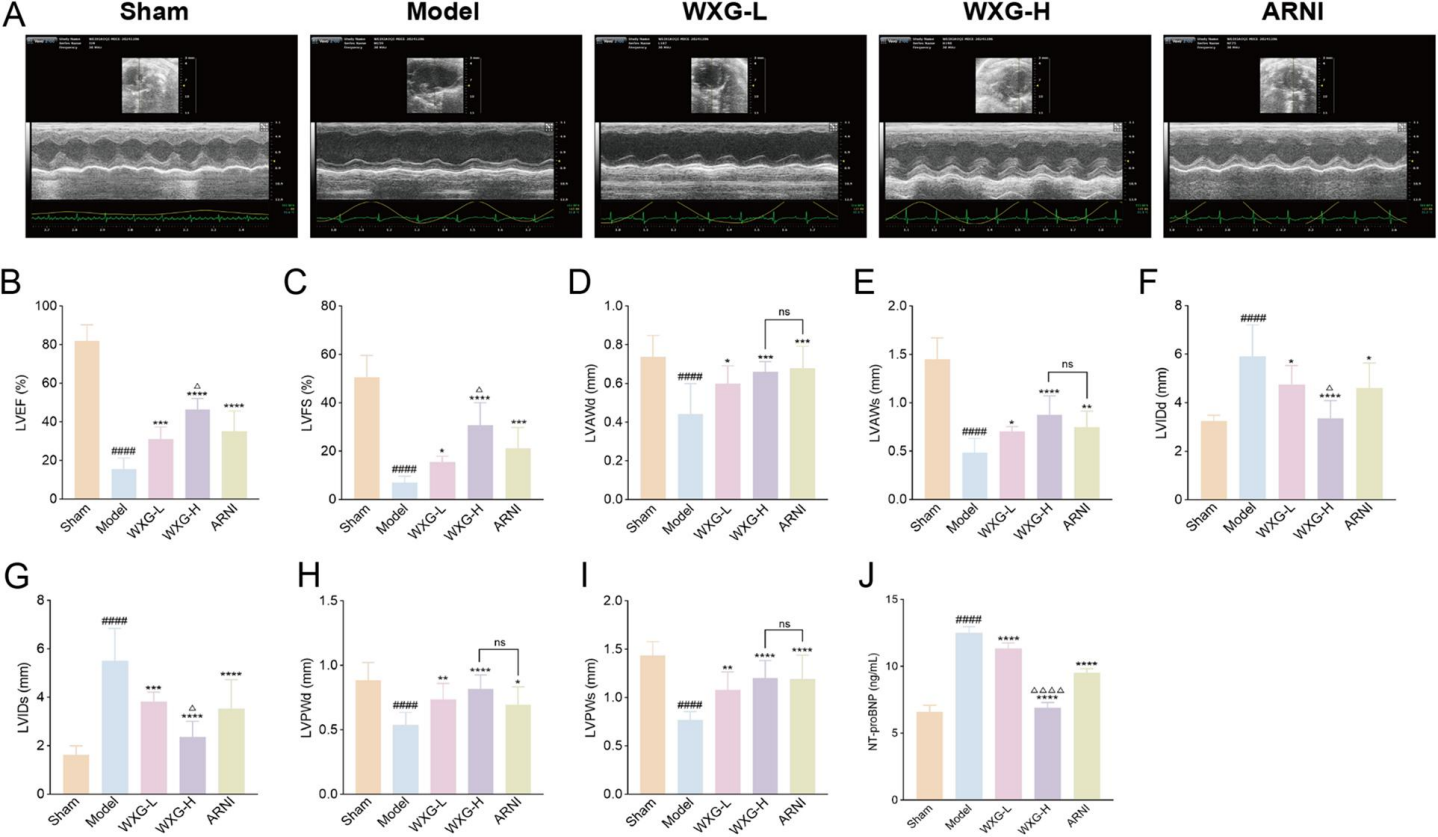
WXG attenuated cardiac dysfunction and structural remodeling in mice with MI-HF. **(A)** Echocardiography of MI-HF mice in 5 group. **(B–I)** The comparison of echocardiographic parameters (LVEF, LVFS, LVAWd, LVAWs, LVIDd, LVIDs, LVPWd, and LVPWs) retrieved among distinct groups of mice (*n* = 10). **(J)** NT-proBNP levels in serum of mice (*n* = 10). ^####^*p* < 0.0001, compared with Sham Group. *p < 0.05, **p < 0.01, ***p < 0.001, ****p < 0.0001, compared with Model Group. ^△^*p* < 0.05, ^△△△△^*p* < 0.0001, compared with ARNI Group. ^ns^*p* > 0.05, compared with ARNI Group.

We constructed MI-HF mice by permanent LAD ligation and assessed the cardiac function of each mouse by high-resolution small-animal echocardiography after 4 weeks treatment with WXG and ARNI (Figure 5A). The results showed that the Model group exhibited significant cardiac dysfunction and structural abnormalities compared with the Sham group. LVEF, LVFS, LVAWd, LVAWs, LVPWd and LVPWs were reduced in the Model group, meanwhile LVIDd and LVIDs were significantly increased. WXG and ARNI significantly attenuated these impairments, with varying degrees of elevation in LVEF, LVFS, LVAWd, LVAWs, LVPWd and LVPWs, and a significant reduction in LVIDd and LVIDs (Figures 5B–I). Also, WXG showed a dose-dependent improvement, with the higher doses of WXG seeming to show even better results, while the lower dose group of WXG had roughly the same effect as ARNI. It is worth mentioning that we also examined NT-proBNP in the serum of mice, which serves as an important indicator for the assessment of HF and an important guide for the determination of cardiac function after MI. The results showed that the higher doses of WXG demonstrated better improvement of NT-proBNP (Figure 5J).

### 3.6 WXG reduces MI-induced cardiac damage and apoptosis

**Figure 6:**
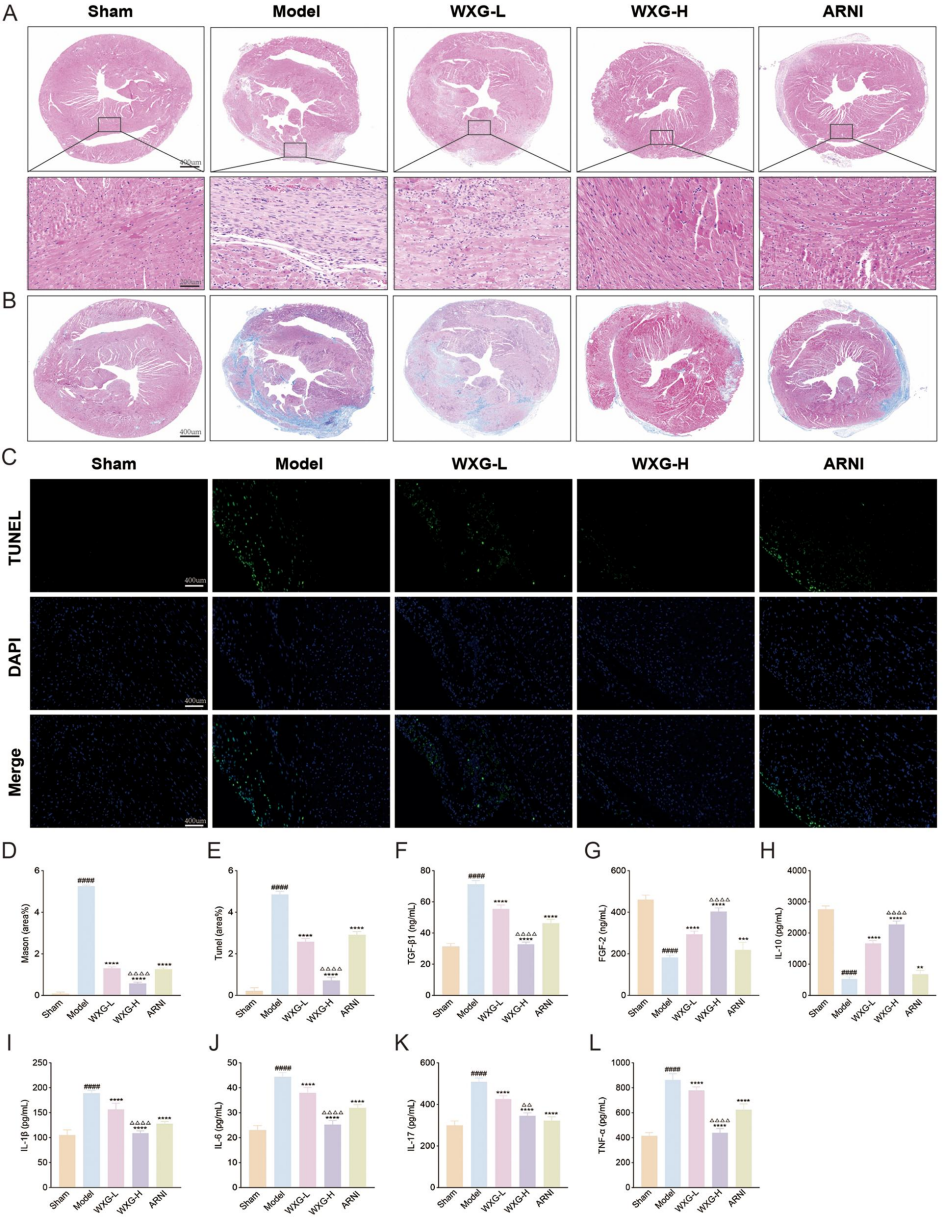
WXG reduces cardiac damage and apoptosis, and ameliorated inflammation in mice with MI-HF. **(A)** HE staining of myocardium at 50× and 200× magnification. **(B)** Mason staining of myocardium at 50× magnification. **(C)** Tunel staining of myocardium at 200× magnification. **(D)** Quantitative assessment of myofibrosis in Masson staining. **(E)** Quantitative analysis of apoptotic cells in Tunel staining. **(F,G)** Serum levels of apoptotic and proliferative cytokines TGF-*β*1 and FGF-2. **(H–L)** Serum levels of inflammatory cytokines IL-10, IL-1*β*, IL-6, IL-17 and TNF-*α* in mice. ^####^*p* < 0.0001, compared with Sham Group. **p < 0.01, ***p < 0.001, ****p < 0.0001, compared with Model Group. ^△△^*p* < 0.01, ^△△△△^*p* < 0.0001, compared with ARNI Group.

To fully investigate the therapeutic potential of WXG, we performed histological staining to analyze its ability to ameliorate cardiac injury. The results of HE staining showed that the Model group exhibited significant myocardial hypertrophy, massive inflammatory cell infiltration and extensive cardiomyocyte necrosis, compared with the Sham group. And the intervention of WXG and ARNI effectively attenuated this myocardial damage and the higher doses of WXG performed better (Figure 6A). The results of Mason staining showed the myocardial myogenic fibers were disarranged and the degree of myocardial fibrosis was more pronounced accompanied by a considerable amount of collagen fiber deposition, compared with the Sham group (Figure 6B). The interventions of WXG and ARNI effectively attenuated the collagen fiber deposition, and the higher doses of WXG performed better (Figure 6D).

Further, in view of the critical role of cardiomyocyte apoptosis in the pathogenesis of MI-HF, we evaluated the effect of WXG on cardiomyocyte apoptosis in MI-HF mice using TUNEL staining (Figure 6C). The results showed that there were more apoptotic cells in the Model group and fewer apoptotic cells in the WXG group and ARNI group and the WXG-H group demonstrated better inhibition of apoptosis (Figure 6E). At the same time, we examined the TGF-*β*1and FGF2 in serum of mice, an important factor regulating myocardial fibrosis and apoptosis. The results showed higher TGF-β1 and lower FGF-2 in the Model group compared to the Sham group. The administered group showed opposite results and WXG-H was superior to WXG-L and ARNI (Figures 6F,G).

### 3.7 WXG ameliorated inflammation in MI-HF mice

Inflammation is a key component of myocardial fibrosis and cardiomyocyte apoptosis. To explore the mechanism by which WXG is involved in this pathway, the serum from MI-HF mice 4 weeks after the drug intervention were collected for inflammatory factor assay. The results showed that the pro-inflammatory factors IL-1β, IL-6, IL-17 and TNF-a were significantly elevated in the Model group, accompanied by a significant decrease in the anti-inflammatory factor IL10, compared with the Sham group. Compared with the Model group, both WXG and ARNI improved the inflammatory state of MI-HF mice to different degrees, and the higher doses of WXG showed better efficacy (Figures 6H–L).

### 3.8 WXG inhibited apoptosis in MI-HF mice through MAPK signaling pathway

**Figure 7:**
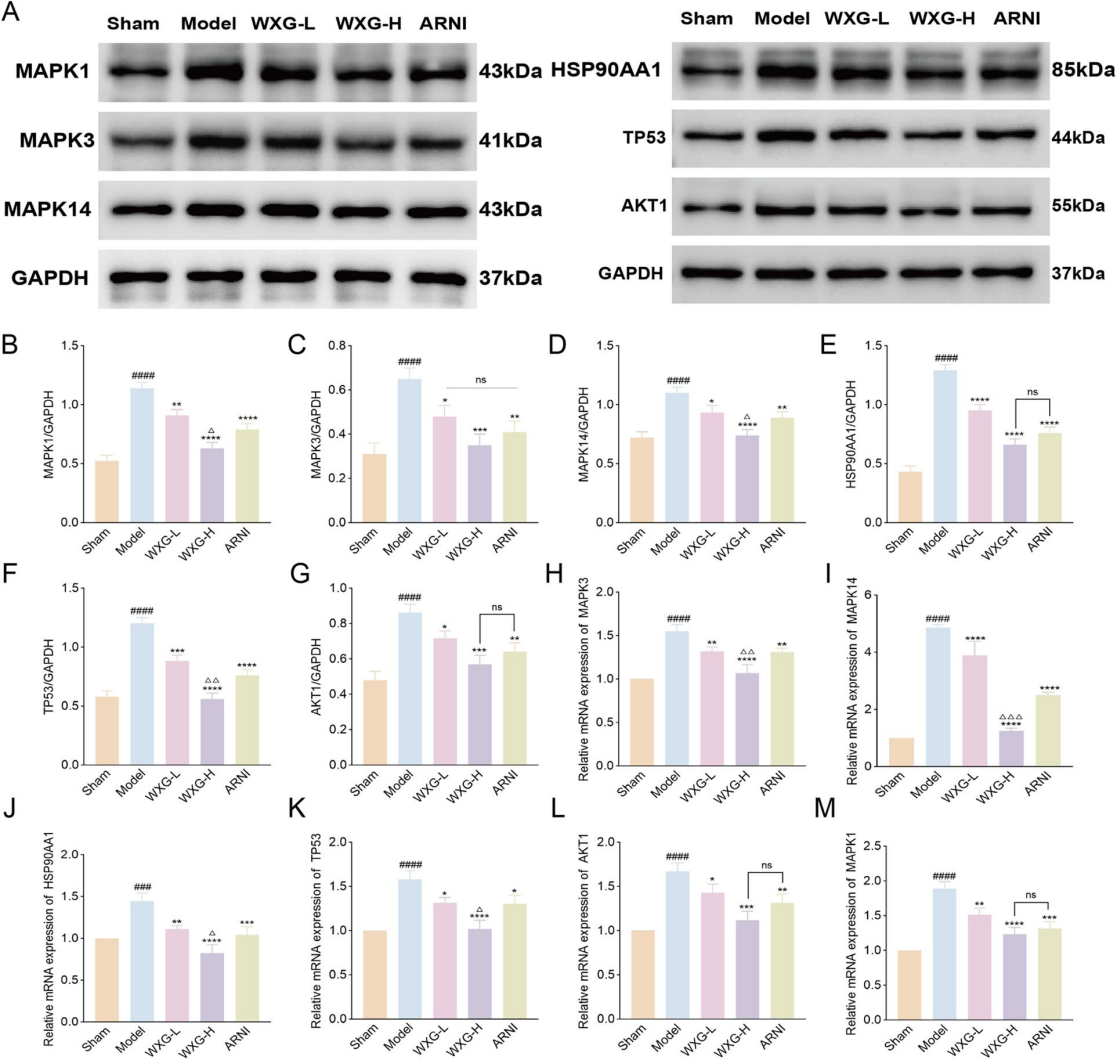
Western blotting and quantitative real-time polymerase chain reaction validation of main targets. **(A)** Proteins expression of MAPK1, MAPK3, MAPK14, HSP90AA1, TP53 and AKT1. **(B–G)** Quantitative evaluation of MAPK1, MAPK3, MAPK14, HSP90AA1, TP53 and AKT1 (*n* = 3). **(H–M)** mRNA expression of MAPK3, MAPK14, HSP90AA1, TP53, AKT1 and MAPK1 (*n* = 3). ^###^*p* < 0.001, ^####^*p* < 0.0001, compared with Sham Group. *p < 0.05, **p < 0.01, ***p < 0.001, ****p < 0.0001, compared with Model Group. ^△^*p* < 0.05, ^△△^*p* < 0.01, ^△△△^*p* < 0.001, compared with ARNI Group. ^ns^*p* > 0.05, compared with ARNI Group.

In this study, WXG better improved the cardiac structure and function of MI-HF mice, but its mechanism of action needs to be further verified. Network pharmacology analysis showed that the efficacy mechanism of WXG was most closely related to the MAPK signaling pathway, and MR confirmed high expression of MAPK3 in HF, a star gene of apoptosis. To further validate our findings, we detected key proteins on the MAPK signaling pathway using WB and RT-qPCR. The results showed that the expression of MAPK1, MAPK3, MAPK14, HSP90AA1, TP53 and AKT1 were highly expressed in the Model group compared to the Sham group, and WXG reduced the expression of these proteins and genes (Figures 7A–M).

**Figure 8:**
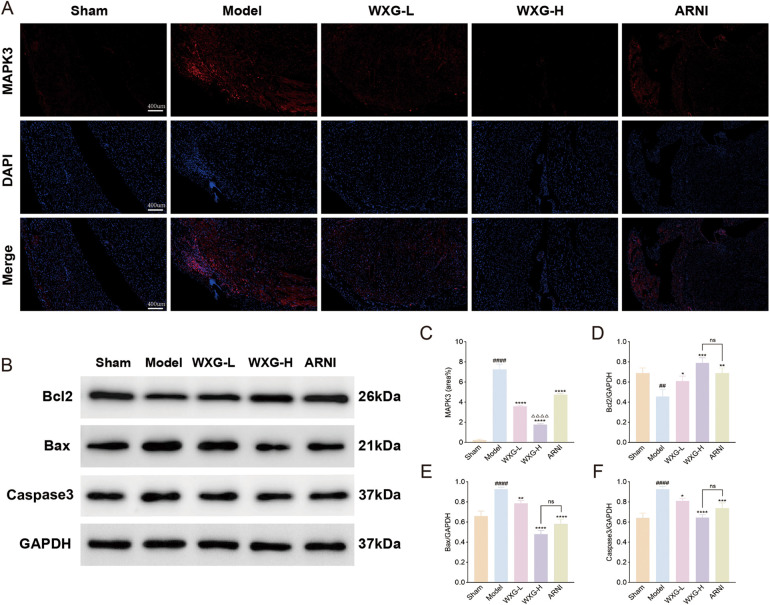
Immunofluorescence and western blotting of apoptosis-related proteins. **(A)** immunofluorescence of MAPK3. **(B)** Proteins expression of Bcl2, Bax and Caspase3. **(C)** Quantitative analysis of MAPK3 in immunofluorescence. **(D–F)** Quantitative evaluation of Bcl2, Bax and Caspase3 (*n* = 3).

In addition, the spatial localization and number of MAPK3 were tracked using immunofluorescence (Figure 8A). The results showed that the hearts of mice in the Model group contained more MAPK3 with a wide range of distribution, whereas WXG group and ARNI group contained less MAPK3. WXG-H showed better results compared to ARNI group (Figure 8C). We also detected the expression of apoptotic proteins downstream of the MAPK signaling pathway (Figure 8B). The results showed lower expression of Bcl2 and higher expression of Bax and Caspase in the Model group compared to the Sham group. Compared with the Model group, WXG and ARNI exhibited higher expression of Bcl and lower expression of Bax and Caspase, and there was no significant difference between them (Figures 8D–F).

## 4 Discussion

Myocardial infarction is a disease caused by acute obstruction of the coronary arteries, leading to insufficient blood supply in myocardial region, which resulting in ischemia and necrosis of myocardium (14). Globally, more than 20 million people die of cardiovascular diseases each year (15), with 50–250 deaths per 100,000 people due to MI, posing a serious threat to people's lives and health (16). Currently, the main focus is on PCI, coronary artery bypass grafting (CABG) and long-term antiplatelet medication for MI. These treatments have greatly reduced mortality in MI, but the incidence of HF after MI has instead increased and, as a result, has significantly increased the long-term risk of death in patients. The study found that about 9.1% patients were hospitalized for HF within 1 year after MI (17). Approximately 20%–30% MI patients develop HF within 5 years (18). Therefore, it's our current research focus to explore the mechanism of MI-HF and find a therapeutic regimen to delay and prevent MI-HF.

Ventricular remodeling is a major cause of MI-HF. Acute occlusion of coronary arteries after MI results in myocardial ischemia and hypoxia, causing persistent changes in size, morphology, structure, and function of the ventricles, which are manifested by left ventricular enlargement, decreased LVEF, and abnormal localized ventricular wall activity (19). When MI occurs, cardiomyocytes are in a state of ischemia and hypoxia, mitochondrial oxidative phosphorylation is impaired, ATP production is reduced, and mitochondrial energy metabolism is impaired. This leads to the release of pro-apoptotic signals, which in turn induces cardiomyocyte apoptosis (20). The loss of cardiomyocytes leads to stretching or compensatory hypertrophy of surviving cardiomyocytes due to increased volume or pressure load. It is accompanied by a disruption of extracellular matrix homeostasis, which leads to a vicious cycle of ventricular remodeling until the onset of HF (21).

Inflammation also plays an important role in the development of ventricular remodeling. When cardiomyocytes are ischemic and hypoxic, the anti-inflammatory signaling is disrupted, leading to a burst of inflammatory factors such as IL-1β, IL-6 and TNF-*α* that drive myocardial injury (22). Under energetic stress, the mitochondria of cardiomyocytes produce large amounts of reactive oxygen species (ROS) to stimulate the activation of NLRP3 and NF-kB, exacerbating the inflammatory response and driving extracellular matrix deposition and structural cardiac changes (23, 24). The directions of our research are to inhibit apoptosis of cardiomyocytes after MI and improve the inflammatory environment.

The pathophysiological mechanisms underlying MI-HF are complex, with traditional understanding focusing on ventricular remodeling driven by “neuroendocrine hyperactivation.” Based on this, modern drugs such as renin-angiotensin-aldosterone system inhibitors and beta-blockers have achieved landmark success (25). However, these therapies primarily target downstream pathological responses, offering limited intervention for the recently identified core mechanism initiating remodeling—persistent chronic low-grade inflammation following MI (26). Moreover, side effects from some drugs often render patients unable to tolerate target doses or even necessitate discontinuation, hindering optimal therapeutic outcomes. For instance, Angiotensin-Converting Enzyme Inhibitor and Angiotensin II Receptor Blocker may cause hypotension, renal impairment, or cough (27); beta-blockers may exacerbate fatigue, bradycardia, or affect glucose and lipid metabolism (28); diuretics may induce electrolyte disturbances (29). Furthermore, even with standardized treatment, many patients persistently suffer from residual symptoms such as palpitations, shortness of breath, extreme fatigue, low exercise tolerance, and night sweats, leading to significantly reduced quality of life (30). Modern drug therapies offer limited improvement for such symptoms, representing a significant unmet need. Traditional Chinese Medicine, which has a long history, is a form of treatment worth investigating (31). As a herbal compound, WXG demonstrated good efficacy in MI with a favorable safety profile. Previous studies have found that WXG could improve the symptoms of angina pectoris, ischemic electrocardiogram, blood rheology and blood lipids. In the study, MI-HF model mice were constructed and fed with WXG for 4 weeks. The results showed that WXG improved cardiac function, decreased inflammation levels, and reduced myocardial fibrosis and cardiomyocyte apoptosis.

Meanwhile, in order to elucidate the unknown components of the herbal compound, we performed LC-MS/MS of the *in vitro* components and drug-containing serum of WXG, and found that WXG mainly contained Astragaloside IV, Paeoniflorin, Liquiritin, Albiflorin, Ononin, and Pratensein 7-O-beta-D-glucopyranoside, among other components. Numerous studies have shown that these components inhibit inflammation and apoptosis to improve MI-HF.

Astragaloside IV, as the main active ingredient of the Astragalus membranaceus, can regulate oxidative stress and pro-fibrotic signaling pathways, inhibit the accumulation of collagen fibers and reduce collagen content to exert anti-myocardial fibrosis effects (32). And it also improves immune and inflammatory states (33). Paeoniflorin was found to ameliorate inflammation and immune dysfunction, exhibiting favorable antithrombotic activity (34, 35). Experimental study demonstrated that Paeoniflorin effectively reduces the level of IL-6 to regulate PARKIN-mediated mitochondrial autophagy and mitochondrial damage (36). Liquiritin, a flavonoid known for its cardioprotective properties, exerts an inhibitory effect on inflammation, mitochondrial damage and apoptosis to Attenuate hypoxia/re-oxygenation-induced cardiomyocyte injury (37). Moreover, Liquiritin could inhibite the expression of CCL5 to improve cardiac function and attenuate oxidative damage and inflammation in MI rats (38). It was shown that Albiflorin, a key active compound in Paeonia lactiflora Pall, protects myogenic cells from oxidative stress-induced DNA damage and cell death by activating Nrf2/HO-1 signaling (39). Albiflorin also reduced the level of biomarkers of myocardial injury in serum, which also provided new insights into myocardial proliferation and anti-apoptosis (40). Ononin inhibits endoplasmic reticulum stress and activates SIRT3 signaling pathway to suppress cardiomyocyte apoptosis (41). Pratensein 7-O-beta-D-glucopyranoside also showed better efficacy in apoptosis and inflammatory responses, inhibiting oxidative stress and NLRP3 inflammatory vesicle activation (42).

In terms of mechanistic studies, we found that WXG may inhibit MI-HF by modulating the MAPK signaling pathway using network pharmacology and bioconfidence analysis. The molecular docking results showed that the core components of WXG bind better to the key proteins on the MAPK signaling pathway, such as MAPK1, MAPK3, MAPK14, HSP90AA1, TP53 and AKT1. To assess the relationship between apoptotic signaling and HF at the genetic level, MR was performed. The results showed that the expression of MAPK3 was significantly associated with HF in a variety of tissues, which reinforces our conjecture. Further, we validated our discovery of apoptotic proteins using WB, RT-qPCR and immunofluorescence. Bax and Bcl-2 play an important role as key regulators in apoptotic signaling pathways. As a key cysteoaspartic enzyme Caspase3 involve in the final stages of apoptosis (43). After receiving apoptotic signals, Bax increases the permeability of the mitochondrial membrane, releases apoptosis-related factors such as cytochrome C into the cytoplasm, and combines with apoptosis protease activating factor-1 (Apaf-1) to activate Caspase 3 and initiate apoptotic programs (44). As apoptosis-suppressing proteins, Bcl-2 protects cells from apoptosis mainly by inhibiting the activity of pro-apoptotic factors such as Bax (45). Our results suggested that WXG regulates the MAPK signaling pathway while inhibiting the expression of Bax and Caspase 3 and promoting the production of Bcl-2 to achieve cardioprotection.

## 5 Future directions

In this study, we explored the mechanism of WXG in the treatment of MI-HF using network pharmacology and MR. The efficacy and mechanistic targets of WXG were also validated in animal experiments. On the downside, multi-omics techniques and cellular experiments were not carried out in this study, which is the next step in our research. Future research may employ *in vitro* models of MI-HF in combination with multi-omics technologies to thoroughly validate the core mechanism by which WXG modulates the phosphorylation dynamics of the MAPK signaling pathway. First, an hypoxia/reoxygenation injury model will be established in H9c2 cardiomyocytes. Following intervention with a drug-containing serum gradient of WXG and specific modulation of the MAPK pathway, phospho-proteomics will be employed for high-throughput screening of phosphorylation sites in core MAPK pathway kinases and key downstream targets of MI-HF. Combined with time-series analysis to track dynamic changes in phosphorylation levels, this approach could elucidate the temporal patterns, dose-dependent relationships, and critical anti-apoptotic time windows of MAPK pathway activation under WXG. Second, WB will be employed to validate expression differences in key phosphorylated proteins and their downstream target proteins. And the immunofluorescence dual-labeling technology could visually demonstrate the association between the subcellular localization of phosphorylated MAPK kinases and cardiomyocyte apoptosis. Additionally, single-cell phospho-flow cytometry can be employed to resolve the heterogeneity of MAPK phosphorylation states within cell populations. By integrating transcriptomics and metabolomics analyses, this approach enables the exploration of molecular networks upstream and downstream of the MAPK pathway regulated by the active components of WXG, as well as the underlying mechanisms of metabolic reprogramming.

## 6 Conclusion

WXG may reduce inflammatory response and enhance cardioprotection and anti-fibrosis by inhibiting the expression of MAPK signaling pathway and its downstream effect proteins. The cardioprotective effects of WXG may be attributed to its core components, including Astragaloside IV, Paeoniflorin, Liquiritin, Albiflorin, Pratensein 7-O-beta-D-glucopyranoside and Ononin.

## Data Availability

The raw data supporting the conclusions of this article will be made available by the authors, without undue reservation.
